# KINECAL: A Dataset for Falls-Risk Assessment and Balance Impairment Analysis

**DOI:** 10.1038/s41597-023-02375-w

**Published:** 2023-09-18

**Authors:** Sean Maudsley-Barton, Moi Hoon Yap

**Affiliations:** Department of Computing and Mathematics, Manchester Metropolitan University, Faculty of Science and Engineering, Manchester, M1 5GD UK

**Keywords:** Quality of life, Population screening

## Abstract

The field of human action recognition has made great strides in recent years, much helped by the availability of a wide variety of datasets that use Kinect to record human movement. Conversely, progress towards the use of Kinect in clinical practice has been hampered by the lack of appropriate data. In particular, datasets that contain clinically significant movements and appropriate metadata. This paper proposes a dataset to address this issue, namely KINECAL. It contains the recordings of 90 individuals carrying out 11 movements, commonly used in the clinical assessment of balance. The dataset contains relevant metadata, including clinical labelling, falls history labelling and postural sway metrics. KINECAL should be of interest to researchers interested in the clinical use of motion capture and motion analysis.

## Background & Summary

Complications associated with injurious falls are the most common cause of death for those aged over 65^1^. In the UK, 1/3 of those aged over 65 and 1/2 of those aged 80 will fall once a year^2^. In addition to the immediate pain and discomfort, even a single fall can lead to a range of associated conditions, such as depression, anxiety and social isolation.

In order to identify those in need of help, a falls-risk assessment must be carried out^2^. However, there is a dichotomy at the heart of falls-risk assessment. Lab-based research tends to use expensive equipment (force plates and marker-based motion capture) to quantify balance impairment. Even here, the costs mean that force plate data tends to dominate. In clinical practice, observational tests are the dominant form of assessment. This means there is difficulty in translating state-of-the-art research into clinical practice. In addition, clinical tests that have shown great utility in falls risk assessment, such as the Sit to Stand x 5 (STS-5) and 3 m walk, are difficult to instrument using a force plate. These tests could be instrumented using marker-based motion capture, but even if one ignores the cost of such systems, the space required and set-up time makes this option impractical for everyday assessment. Markerless motion capture could provide a practical solution to bridge the gap between research and practice. Markerless solutions can provide joint angles, akin to those derived from marker-based solutions, and sway metrics, akin to those derived from force plates. These systems are not without issues, but they can provide insight, difficult to achieve any other way, away from the lab. However, research into their use as an objective method of assessing balance, frailty and falls-risk is under-researched. As is often the case, there is a chicken and egg situation when it comes to appropriate data on which to base the development of useful methods of assessment using these devices. The proposed dataset, named *KINECAL* addresses this need.

Kinect, and more generally Red Green Blue + Depth (RGB + D) data, is used extensively in human action recognition (HAR) research^3^. This area of research has made huge strides in recent years, driven forward by the availability of a diverse range of publicly available datasets^4–6^. On the face of it, it would seem this type of dataset would be useful in the clinical study of human movement. However, the focus is quite different. The purpose of HAR, is to identify a small set of human movements (actions) from a nearly infinite set of possibilities. The clinical use of motion capture seeks to quantify the quality of a prescribed set of movements. In addition, anonymised clinically-important metadata such as age, height, weight, and meaningful labels of impairment are rarely included in HAR datasets.

Firman^4^, in his review of RGB + D datasets, detailed 45 datasets, 44 of which were recorded using Kinect. Of these datasets, only one, the K3Da dataset^7^ had movements that are useful in the assessment of balance disorders, it has some metadata, but it is limited to age, height and weight.

Since the publication of the Firman paper^4^, a few datasets have been created to address these issues. The Multimodal Dataset^8^ contains clinically relevant movements (Timed get-up-and-go (TUG), a single 30 second chair stand, a 45 second unilateral stance, and a 2 minute step test). However, the dataset comprises just 21 subjects, evenly split between young and old, and none of the participants had known movement impairment at the time of testing. The KIMORE Dataset^9^, has 78 subjects, 44 healthy (mean age 36.7 ± 16.8 years) and 32 suffering from motor dysfunction (mean age 60.44 ± 14.2 years). The dataset includes clinical scoring of the movements. However, the movements do not relate directly to falls-risk. Table 1 provides a comparison between KINECAL and other datasets, containing clinical movements.

**Table 1:** A comparison of KINECAL and previous datasets that contain clinically significant movements.

	Multimodal Dataset	K3Da	KIMORE	KINECAL
	8	7	9	
Number of participants	21	54	78	90
Depth data	✗	✓	✓	✓
Skeleton data	✓	✓	✓	✓
Clinical evaluation	✗	✗	✓	✓
Falls related labels	✗	✗	✗	✓
Postural Sway Metrics	✗	✗	✗	✓
Recordings in informal settings	✗	✗	✗	✓

KINECAL contains the recordings of 90 participants carrying out 11 movements, commonly used in the clinical assessment of balance impairment, frailty and falls-risk. Details of how each movement was carried out are shown in Table 2. The participants are grouped by both age and falls-risk. This enables researchers to disambiguate factors which relate to each.

**Table 2:** A description of movements in the recording of  KINECAL, and how they were described to the participants.

	Standard instruction to the participants
**STS-5**	From a seated position, with your arms crossed over your chest, rise extending your legs fully, then sit down again. Repeat five times, as quickly as possible.
**Quiet standing,** **Eyes open,** **Firm surface**	Stand feet close together, eyes open and arms by your side. The test is terminated after 20 seconds, or if lost balance.
**Quiet standing,** **Eyes closed,** **Firm surface**	Stand feet close together, eyes closed and arms by your side. The test is terminated after 20 seconds, or if lost balance.
**Quiet standing,** **Eyes open,** **Foam**	Same instructions as for standing on a firm surface, but perform on a foam.
**Quiet standing,** **Eyes closed,** **Foam**	Same instructions as for standing on a firm surface, but perform on a foam.
**Semi-tandem Stance**	Stand with the toe of the back foot against the side of the heal of the front foot. Ether foot can be forward, which ever is most comfortable. The test is terminated after 20 seconds.
**Tandem Stance**	Stand with the toe of the back foot against the back of the heal of the front foot. Ether foot can be forward, which ever is most comfortable The test is terminated after 20 seconds.
**Unilateral stance,** **Eyes open**	Stand on one leg, whichever is most comfortable, with the other leg flexed 6 inches off the ground, hands by your side, eyes open. The test is terminated after 20 seconds or when the lifted leg touches the ground.
**Unilateral stance,** **Eyes closed**	Stand on one leg, whichever is most comfortable, with the other leg flexed 6 inches off the ground, hands by your side, eyes closed. The test is terminated after 20 seconds or when the lifted leg touches the ground.
**TUG**	From a seated position, stand, and walk to a marker 3 m away and return to the seat
**3 m walk**	From a standing position, walk to a marker 3 m away, and return to the seat

## Methods

### Participants

90 participants, aged from 18 to 92, were recorded for the KINECAL dataset, carrying out a range of movements commonly used in clinical tests. The recordings were made using a Kinect V2. Further details of data collection are shown below. Ethical approval was obtained from the Manchester Metropolitan University Research Ethics Committee (ethics approval ref: 020517-ESS-CC(1), and ref: SE161757). All participants provided written informed consent. Participants were excluded if they had any of the following:

- Treatment for cancer in the previous 2 years
- Joint replacement in the previous year
- Broken a leg, or hip bone or had a joint replaced, e.g. hip or knee, in the previous 2 years
- Any lower limb amputation
- Suffering from neuromuscular conditions (e.g. multiple sclerosis)
- Have been diagnosed with Alzheimer’s or Dementia
- Cannot read and communicate in English, either verbal or written

### Clinically significant movements

Table 2 provides a list of the clinically significant movements included in the dataset, along with the instructions given to each participant. The movements are: Quiet standing, eyes open (EO) and eyes closed (EC), semi-tandem stance EO, tandem stance EO, 3 m walk and 5 STS-5, (this constitutes the Short Physical Performance Battery (SPPB) assessment). To assess a participant’s ability to react to perturbation, the quiet standing trials EO/EC were repeated while standing on a compliant surface (Airex balance pad). Finally, two movements which are often used as standalone tests are included, i.e. TUG and unilateral stance EO/EC.

### Data collection

**Fig. 1:**
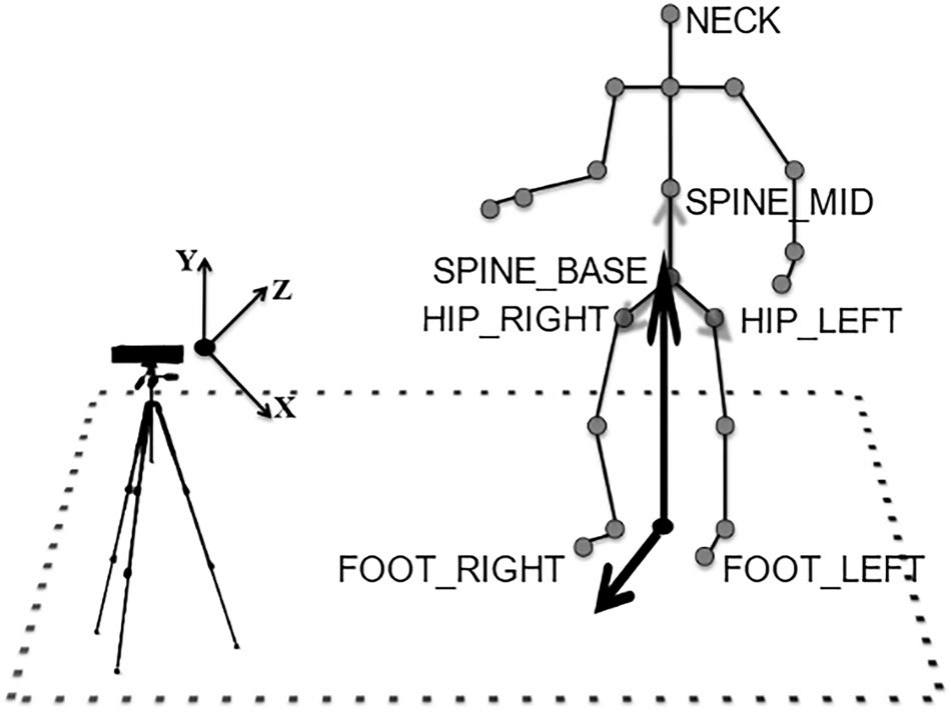
The relationship between an individual being recorded and the Microsoft Kinect camera. The CoM is calculated as the Euclidean average of the *HIP_LEFT*, *HIP_RIGHT* and *SPINE_MID* joints.

**Fig. 2:**
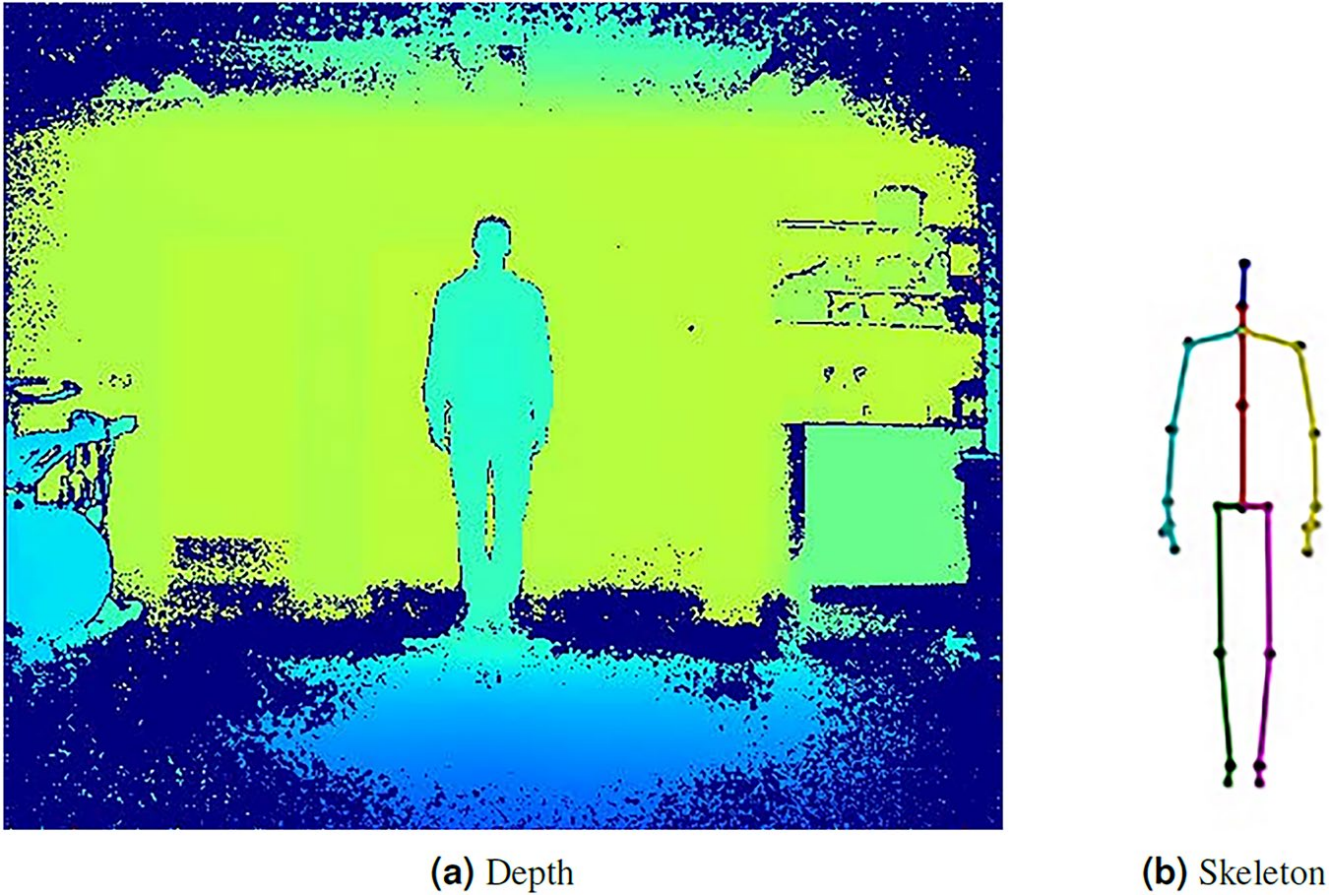
Kinect point-of-view This figure is of a young participant in quiet stance. (**a**) is a depth image, (**b**) is the 25-joint skeleton created by Kinect.

Recordings were made using a Kinect camera mounted on a tripod at a height of 1.14 m from the ground plane. Participants were asked to stand 3 m from the camera for static stances and 4 m for TUG, and 3 m walk (the additional meter was needed to capture the entire 3 m walk). Participants were recorded using custom software, skel recorder, written using Visual Studio 2015 and the Kinect SDK 2.0. skel recorder captures Depth and Skeleton data and stores it to hard disk. Figure 1 shows the equipment setup and Fig. 2 shows the output from the Kinect. This image was taken in laboratory conditions, but other recordings in KINECAL were made in more informal settings, such as church halls.

### Quality control

Kinect can miss-classify structures, such as tables, which are in the frame along with the participants. If this happens, aberrant and truncated skeletons are included in the recordings. These types of artefacts reduce the quality of the dataset. In addition, some of the initial recordings were started after the movement had begun or stopped before the movement was completed. To ensure only good-quality recordings were included in the dataset, videos were created from the depth and skeletal data. These videos were reviewed manually. Any recordings with issues were excluded from the dataset.

### Dataset structure

In designing KINECAL, previous studies relating to ageing and falls-risk were reviewed. Some studies considered just age-related changes, e.g. young ( < 35 years old) vs older (≥65 years old) populations^10–12^. This polarisation is helpful if the question is, how does postural sway change with age. However, using this type of data alone can be problematic when applied to falls-risk. While ageing plays a part in falls-risk, it is not the whole story. For example, if we consider master athletes (individuals over the age of 30, but many of whom are aged over 80, who still take part in athletic competition). In spite of demanding regimes of training and competition, the occurrence of falls for master athletes was found to be no higher than it was for younger athletes^13^. While they are seen as functionally fitter when compared to those of a similar age who do not compete in sporting events, the effects of ageing are still detectable as changes in motor neurons^14,15^, but this does not translate to an increase in the number of falls. McPhee *et al*.^16^ suggested that the protective effects of regular exercise are universal and not limited to elite athletes. An alternative approach, when considering falls-risk, is to control for age. This is the approach taken by^17–19^, who compared aged-matched populations of older fallers and non-fallers. However, this approach can make it just as challenging to understand the interaction of age and physical function.

With these factors in mind, KINECAL is structured to help aid the separation of falls-risk from age effects. The following section details how participants were labelled to provide a range of groupings when working with the data.

#### Self-reported labels

All participants were asked the following question. “**Have you had any fall,** **including a slip or trip in which you lost your balance and landed on the floor or ground or lower level in the past 12 months?**”^20^. Possible answers were [**None,** **One,** **Two,** **Three,** **Four or more**].

Based on the answer they gave and their age, participants were split into the following groups:

- **Healthy-Adult**, members of this group, were all <65 years old and gave the answer **None**
- **Non-Faller**, members of this group, were all ≥65 years old and gave the answer **None**
- **Self-reported-Faller**, members of this group, were all ≥65 years old and gave the answer **One,** **Two,** **Three,** **Four or more**

Table 3 details the numbers in each group, along with their age range and the gender split.

**Table 3:** Grouping of Self-reported Labels: This table shows the split between the different groups in terms of mean age, total numbers and gender split.

	Description	Age (±95% CI)	Total Number	Male	Female
Healthy-Adult	<65 years, no history of falls in the last 12 months	mean age 46.2 (±22.7)	33	22	11
Non-Faller	≥65 years, no history of falls in the last 12 months	mean age 73.3 (±11.7)	33	16	17
Self-Reported-Faller	≥65 years, reported ≥1 falls in the last 12 months	mean age 72.6 (±13.6)	24	15	9

A description of each group is also included.

#### Single and multiple fallers

The **Self-reported-Faller** group can be further split into single and multiple fallers. We make this distinction because someone who answered **one** to the falls history question might simply be the victim of bad luck and not someone with a high likelihood of future falls. Someone who declared they have fallen multiple times is more likely to suffer future falls and could be thought of as a true faller^21^. It is up to the individual researcher to make the best use of these labels.

- **Self-reported-Faller_s**, members of this group, were all ≥65 years old and gave the answer **One**
- **Self-reported-Faller_m** members of this group, were all ≥65 years old and gave the answer **Two,** **Three,** **Four or more**

Table 4 details the numbers in each group, along with their age range and the gender split.

**Table 4:** Grouped Sub-labelling of Self-reported-Fallers: This table shows the split between the different single (_s) and multiple fallers (_m), in terms of mean age, total numbers and gender split.

	Description	Age (±95% CI)	Total Number	Male	Female
Self-reported-Faller_s	≥65 years, reported 1 fall, in the last 12 months	mean age 72.3 (±15)	15	7	8
Self-reported-Faller_m	≥65 years, reported > 1 fall, in last 12 months	mean age 73.1 (±11.7)	9	2	7

A description of each group is also included.

*Note: the**** Self-reported-Faller_s**** and**** Self-reported-Faller_m**** groups are made up of members of the**** Self-Reported-Faller**** group. Participants are multipally labelled, which provides flexibility in use. However, it is up to the individual researcher to decide how to best use these labels*.

#### Clinical labelling

The movements recorded in the dataset provide an alternative means of labelling participants. Moreover, one which relates to clinical tests of physical impairment, hence we call this clinical labelling.

Clinical labelling was a two-stage process. (1) The recordings for each participant were played back and marked against accepted thresholds for physical impairment and falls-risk, for each of the following tests: SPPB^22^; Slow 3 m walk^23^; TUG^24,25^ and slow time to complete STS-5^26^. (2) Any individual categorised as impaired for a least two of these tests was labelled **Clinically-At-Risk** in the dataset. Using this method, six people were identified as belonging to the **Clinically-At-Risk** group. Details of this group are shown in Table 5. The thresholds of impairment for each test are discussed below.

**Table 5:** Details of the Clinically-At-Risk group: mean age, total numbers and gender split are shown.

	Description	Age (±95% CI)	Total Number	Male	Female
Clinically-At-Risk	≥65 years identified as impaired by ≥2 clinical tests	mean age 80.3 (±11.8)	6	1	5

*Note: the**** Clinically-At-Risk**** group is made up of members of the**** Non-Faller**** group (2) and**** Self-Reported-Faller**** group (4)*. *Participants are multipally labelled*, *which provides flexibility in use*. *However*, *it is up to the individual researcher to decide how to best use these labels*.

### Thresholds of clinical-impairment

This section details the thresholds applied to each clinical test.

#### SPPB

The SPPB test was carried out using the protocol described in^27^ and scored based on the scoring system shown in Table 6. The participant was classified using the criteria detailed in Table 7. Those who were classified as having **Moderate Limitations** or **Severe Limitations** were marked as at-risk for this test.

**Table 6:** SPPB Scoring.

Test	Score
**Quiet Standing**	
Held for 10 sec	1
Not Held for 10 sec	0
Not Attempted	0
**Semi-Tandem Stance**	
Held for 10 sec	1
Not Held for 10 sec	0
Not Attempted	0
**Tandem Stance**	
Held for 10 sec	2
Held for > 3 sec < 10 sec	1
Held for < 3 sec	0
Not Attempted	0
**3 m Gait Speed**	
Unable to complete or > 1 minute	0
> 6:31	1
> 4:40 < 6:31	2
> 3:37 < 4:40	3
< 3:37	4
**STS-5**	
Unable to complete or > 1 minute	0
> 16:42	1
> 13:42 < 16:42	2
> 11:12 < 13:42	3
< 11:12	4

**Table 7:** SPPB classification.

Score	Classification
0–3	Severe Limitations
4–6	Moderate Limitations
7–9	Mild Limitations
10–12	Normal

#### 3 m walk

Quach *et al*.^28^ used a 3 m walk to assess falls-risk in an 18-month-long, longitudinal study of 764 community-dwelling older people, mean age: 78 ± 5 years. They concluded that a walking speed of <0.6 m/s was associated with an increased risk of falling inside. This is equivalent to a time of >5 seconds to complete. Applying this threshold to the 3 m walks in KINECAL, anyone who took >5 seconds to complete the 3 m walk was marked as at-risk, for this test.

### STS-5

Another longitudinal study was undertaken by Ward *et al*.^26^. Over 4 years, they studied 755 community-dwelling older people, mean age: 78.1 ± 5.4 years. The study looked at SPPB as a predictor of injurious falls. The conclusion was that the STS-5 alone was enough to assess falls-risk. They suggested that a time to complete ≥16.7 seconds may be sufficient to identify those at risk of future falls. Applying this threshold to KINECAL, anyone who took >16.7 seconds to complete the STS-5 test was marked as at-risk, for this test.

#### TUG

Shumway-Cook *et al*.^25^ found TUG to be a powerful test for fallers. They used a population of 30 people, split evenly between two groups, i.e. **Non-Fallers** (no historic falls in the last 6 months) with mean age: 78 ± 6 years, and **fallers** (≥2 falls in the last 6 months by history) with mean age: 86.2 ± 6 years. They suggested that those who take >14 seconds to complete the TUG are at **elevated risk of falls**. The Mc Kinly Laboratory^29^ provides a reference for normative scores for TUG, synthesised from the Shumway-Cook paper and 3 more^24,30,31^. This reference extends the recommendations of Shumway-Cook *et al*.^25^, as presented in Table 8. In spite of the other work, the value presented in^25^ remains the current recommendation to identify those at **elevated risk of falls**. Applying this threshold to KINECAL, anyone who took >14 seconds to complete the STS-5 test was marked as at-risk for this test.

**Table 8:** TUG classification.

Time	Classification
30 seconds	Problems, cannot go outside alone, requires gait aid
20 seconds	Good mobility, can go out alone, mobile without gait aid
14 seconds	Elevated risk of falls
10 seconds	Normal

#### Filtering

As with any system that digitises real-world data, Kinect’s signal is bound up with noise, which can be seen as jittering of the skeletal joints. To address this issue, the recordings were filtered using a Butterworth fourth-order zero-lag filter with a cut-off frequency of 8 Hz. The cut-off frequency was calculated using residual analysis of the filtered and unfiltered signal as a function of the filter cut-off frequency, as described by Winter^32^. 8 Hz provided the best balance between the signal distortion and the amount of noise allowed through.

#### Pose normalisation

Each skeleton frame was aligned to the first frame of the recording, making all subsequent movements relative to this initial position^33^, using Eq. 1.

1 $${p}_{n,i}{(x,y,z)}^{* }={P}_{n,i}(x,y,z)-{P}_{0,SPINE\_BASE}(x,y,z)$$

where *p*_*n*,*i*_(*x*, *y*, *z*)* represents the normalised position of the *x*,*y*,*z* axis of joint *i* in frame *n*. ${P}_{0,SPINE\_BASE}(x,y,z)$ represents the position of the *SPINE_BASE* joint in the first frame of the recording, and *P*_*n*,*i*_(*x*, *y*, *z*) represents the positions of joint *i* in frame *n*.

#### Estimation of CoM

**Fig. 3:**
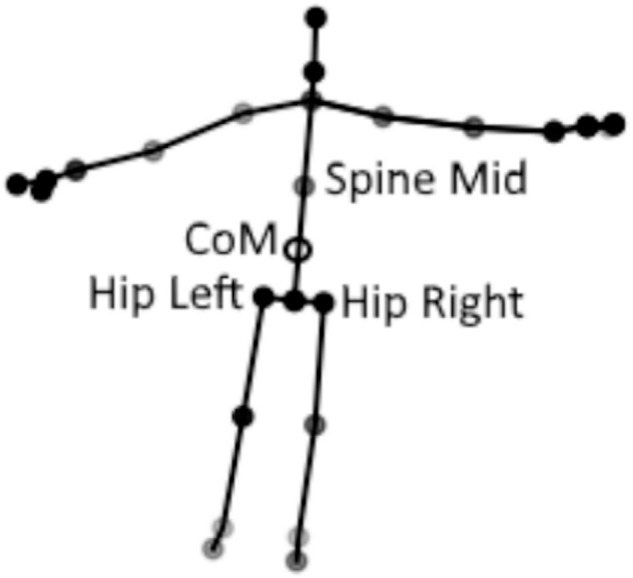
Kinect V2 Skeleton: the joints used to estimate the CoM are labelled, along with the estimate CoM position.

In posturography, postural sway relates to the change in the position of the body’s CoM over time. The most commonly used device for posturography is a force plate. When using force plates, CoM position is estimated from the Centre of Pressure (CoP) at the surface of a force plate^34^. Using the inverted-pendulum model, the position of the CoM is related to the position of the CoP by applying an offset related to an individual’s height^35^. In our work, we derive CoM using a method described in our previous work^36^. It calculates CoM to be the 3D Euclidean mean of 3 joints of the Kinect skeleton: Hip left, Hip right, and Spine mid, as described in Eq. 2. This method has been validated to be equivalent to the CoM derived from the Balance Master in a previous paper^36^.

2 $$\begin{array}{ccc}Co{M}_{x} & = & \frac{HIP\_LEF{T}_{x}+HIP\_RIGH{T}_{x}+SPINE\_MI{D}_{x}}{3}\\ Co{M}_{y} & = & \frac{HIP\_LEF{T}_{y}+HIP\_RIGH{T}_{y}+SPINE\_MI{D}_{y}}{3}\\ Co{M}_{y} & = & \frac{HIP\_LEF{T}_{z}+HIP\_RIGH{T}_{z}+SPINE\_MI{D}_{z}}{3}\\ CoM & = & \left[Co{M}_{x},Co{M}_{y},Co{M}_{z}\right]\end{array}$$

where *HIP_LEFT*, *HIP_RIGHT*, *SPINE_MID* are Kinect joints, indicated on Figure 3. The Figure also shows the resultant position of the CoM for a single frame.

### Generation of sway metrics

The 3D CoM time series is a useful asset for researchers, as is the full depth and skeleton data. However, a set of common force plate metrics has been included for all upright stances. In doing this, the dataset simulates the type of lab-based metrics you would obtain from a force plate. Hence in one dataset, you have clinical and lab-based tests for each participant.

**Fig. 4:**
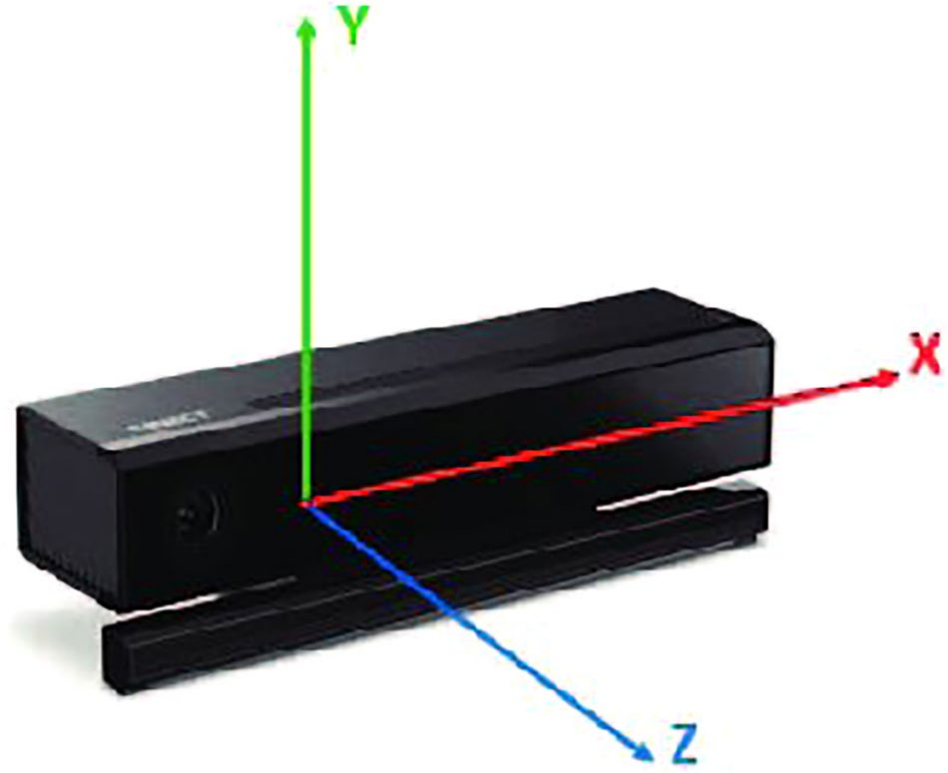
Kinect coordinate system: z-axis projects from the depth camera, with x and y orthogonal to it. This image is recreated from here.

Generally, when working with sway metrics derived from force plates, the CoM position is expressed in only two dimensions, i.e. the anatomical directions Anterior-Posterior (AP) and Mediolateral (ML). Kinect recordings are 3-dimensional, using a right-handed worldview. The origin is the centre of the camera, as shown in Fig. 4. To align the two views, the recordings were first normalised, and then the z-axis of Kinect was mapped to movement in the AP direction of the person being recorded. Similarly, the x-axis was mapped to movement in the ML direction. For this application, the y-axis was ignored.

Table 9 shows the abbreviations used for each metric, alongside a description of each metric. The abbreviations follow the form laid out in Prieto *et al*.^10^.

**Table 9:** Table of sway metrics: This table details the sway metrics from the KINECAL dataset.

	Description
**MDIST**	Mean distance of the RD time series
**RDIST**	RMS distance of the RD time series
**MVELO**	Mean velocity of the RD time series
**TOTEX**	Total excursions of the RD time series
**MFREQ**	Mean frequency of the RD time series
**MDIST_AP**	Mean distance of the AP time series
**RDIST_AP**	RMS distance of the AP time series
**MVELO_AP**	Mean velocity of the AP time series
**TOTEX_AP**	Total excursions of the AP time series
**MFREQ_AP**	Mean frequency of the AP time series
**MDIST_ML**	Mean distance of the ML time series
**RDIST_ML**	RMS distance of the ML time series
**MVELO_ML**	Mean velocity of the ML time series
**TOTEX_ML**	Total excursions of the ML time series
**MFREQ_ML**	Mean frequency of the ML time series
**AREA_CE**	95% Confidence Area

Note. RD refers to the Resultant Distance, AP refers to Anterior-posterior, and ML refers to Mediolateral directions, details of how these metrics were calculated can be found in section 3.

### Sway metric - time series

Force plates often have marks on their surface proscribing foot positions or will automatically centre the recordings. In the case of the recordings presented here. Centring was achieved by subtracting the mean CoM position, for an entire recording, from the CoM value at each time step. This was done separately for movement in the AP and ML directions using Eq. 4. The mean position was calculated using Eq. 3.

The output of Eq. 4 were concatenated to produce the *AP* and *ML* time series. A third time series was calculated, which takes into account movements in the AP and ML directions in a single value. Known as the resultant distance time series (*RD*), it was calculated as the vector distance from the mean CoM position to a pair of points in the AP and ML time series, at each time step. Values for each time step were generated using Eq. 5 and then concatenated to produce RD. Each of the calculated sway metrics was calculated using all three time series.

3 $$\begin{array}{ccc}ML\_\bar{r}a{w}_{i} & = & \frac{1}{n}\mathop{\sum }\limits_{i=i}^{n}ML\_ra{w}_{i}\\ AP\_\bar{r}a{w}_{i} & = & \frac{1}{n}\mathop{\sum }\limits_{i=i}^{n}AP\_ra{w}_{i}\end{array}$$

4 $$\begin{array}{ccc}A{P}_{i} & = & AP\_ra{w}_{i}-AP\_\bar{r}aw\\ M{L}_{i} & = & ML\_ra{w}_{i}-ML\_\bar{r}aw\end{array}$$

5 $$R{D}_{i}=\sqrt{{\left(M{L}_{i}\right)}^{2}+{\left(A{P}_{i}\right)}^{2}}$$

**Fig. 5:**
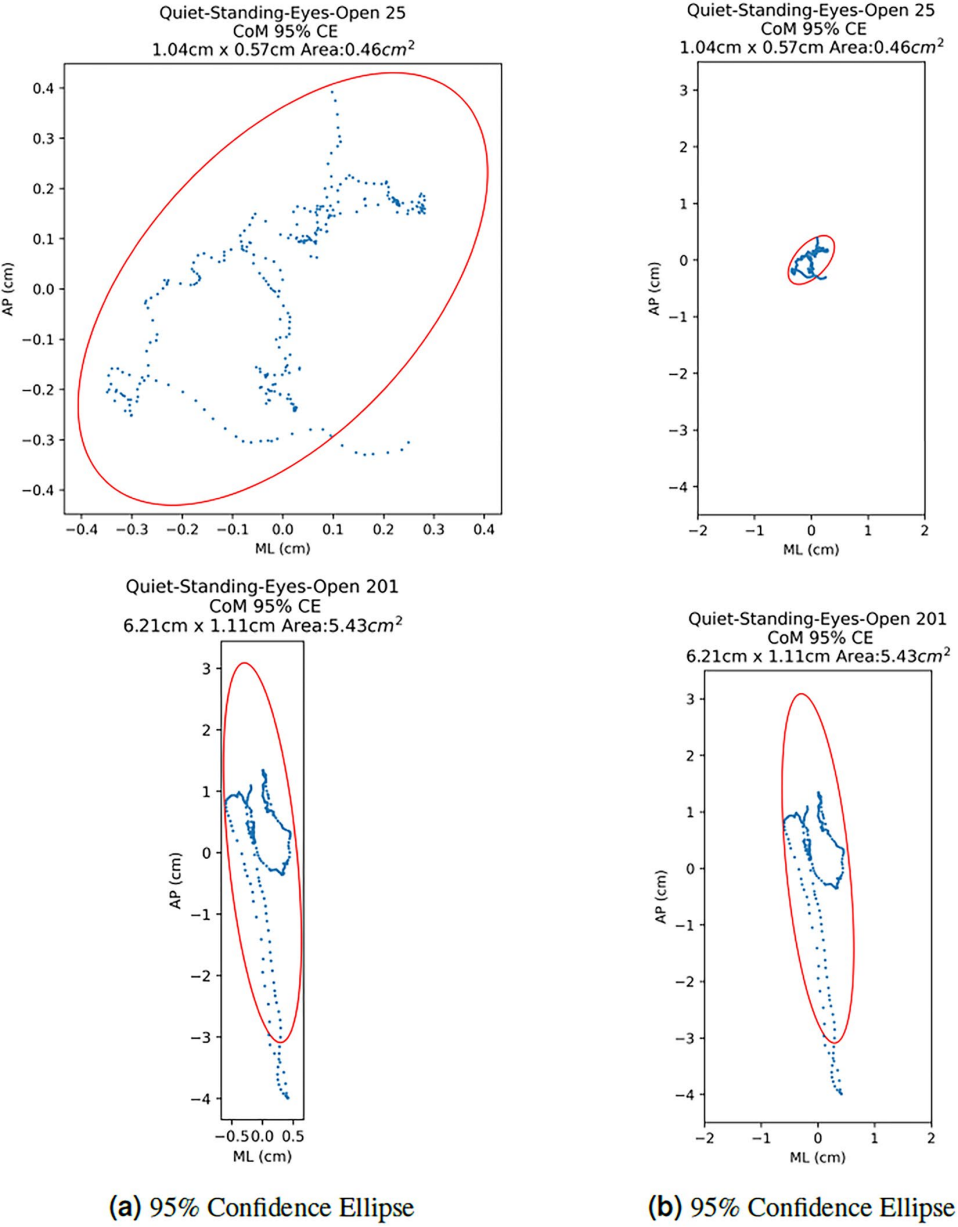
95% Confidence Ellipse and CoM path: This figure compares CoM paths and associated 95% Confidence Ellipses of a person labelled **Healthy-Adult** (25) to someone labelled **Clinically-Impaired** (201), Standing Quietly. Column (a) shows a tight view. Column (b) shows the 95% Confidence Ellipses plotted on the same scale. Note the majority of the increase in the area, for the Clinically-Impaired individual, come from movement in the AP direction.

If the *AP* is graphed against *ML*, the CoM path for each recording is revealed. Figure 5 compares the CoM paths of members of the **Healthy-Adult** and **Clinically-Impaired** groups Standing Quietly with eyes open (blue line). The 95% confidence ellipse is also shown (red ellipse) The calculation of the 95% confidence ellipse is detailed below.

### Calculation of sway metrics

The following section gives details of the equations used to calculate the sway metrics. In these equations, _AP relates to the AP time series (*AP*). For the most part, these equations were also used to calculate the metrics in the ML and RD directions. The exceptions being MFREQ and the 95% confidence ellipse.

### Mean distance of CoM

The most straightforward metric to understand, and to calculate, is the mean distance of the CoM. This is simply the mean, absolute distance moved, from the mean position of the CoM over the time of each trial. Equation 6 was used to calculate MDIST_AP

6 $$MDIST\_AP=\frac{1}{n}\sum \left|A{P}_{i}\right|$$

where *AP* is the AP time series, *i* is a single time step and *n* is the total number of times steps, in the time series. *ML* was used in place of *AP*, to calculate the MDIST_ML. *RD* was used in place of *AP*, to calculate the MDIST.

### RMS distance

The root mean squared process removes the sign and gives more prominence to values further away from the mean CoM position. Equation 7 was used to calculate RDIST_AP.

7 $$RDIST\_AP=\sqrt{\frac{1}{n}\sum A{P}_{i}^{2}}$$

where *AP* is the AP time series, *i* is a single time step and *n* is the total number of times steps, in the time series. *ML* was used in place of *AP*, to calculate the RDIST_ML. *RD* was used in place of, *AP* to calculate the RDIST.

### Total excursion (CoM Path Length)

Total excursion is calculated by summing the distance between successive time steps. This is also known as the CoM path length. Equation 8 was used to calculate TOTEX_AP.

8 $$TOTEX\_AP=\sum \left|A{P}_{i+1}-A{P}_{i}\right|$$

where *AP* is the AP time series and *i* is a single time step. *ML* was used in place of AP to calculate the TOTEX_ML. *RD* was used, in place of *AP*, to calculate the TOTEX.

### Mean velocity

Mean velocity is the total excursion divided by the time for the trial, in seconds. Equation 9 was used to calculate the MVELO_AP.

9 $$MVELO\_AP=TOTEX\_AP/t$$

where *AP* is the AP time series and *t* is the time for the trial in seconds. *ML* was used in place of AP to calculate the MVELO_ML. *RD* was used, in place of, *AP* to calculate the MVELO.

### Mean angular frequency AP and ML

MFREQ_AP is the frequency, in Hz, of a sinusoidal oscillation with an average value of the mean *MDIST_AP* and a total path length of *TOTEX_AP*. This was calculated using Eq. 10.

10 $$MFREQ\_AP=\frac{MVELO\_AP}{4\sqrt{2MDIST\_AP}}$$

where *MVELO_AP* is defined by Eq. 9 and *MDIST_AP* is defined by the Eq. 6. *MVELO_ML* was used in place of *MVELO_AP* and *MDIST_ML* was used in place of *MDIST_AP* to calculate the MFREQ_ML.

### Mean angular frequency

MFREQ is the mean angular frequency, in Hz. This is the number of revolutions per second of the CoM if it had travelled the total excursion around a circle with a radius of the mean distance. It is calculated using values derived from *RD*, given by Eq. 11.

11 $$MFREQ=\frac{MVELO}{2\pi MDIST}$$

where *MVELO* is defined by Eq. 9 and *MDIST* is defined by Eq. 6.

### 95% confidence elliptical area

The AREA_CE is given by the set of equations below (12). The elliptical area is an estimate of the area described by the maximum and minimum AP and ML values of the time series. To reduce the effect of rapid changes in direction, this value is scaled to 1.96 standard deviations of the mean value.

12 $$\begin{array}{ccl}p & = & \frac{co{v}_{AP,ML}}{{\sigma }_{AP}{\sigma }_{ML}}\\ A{P}_{r} & = & \sqrt{1-p}{\sigma }_{AP}1.96\\ M{L}_{r} & = & \sqrt{1+p}{\sigma }_{ML}1.96\\ area & = & \pi A{P}_{r}M{L}_{r}\end{array}$$

where *p* is the Pearson correlation coefficient, *ML*_*r*_ is the ML elliptical radius scaled to the 95% Confidence Interval (CI), and *AP*_*r*_ is the AP elliptical radius scaled to the 95% CI.

## Data Records

The raw data files and the calculated sway metrics are stored in a physionet repository^37^. The section below provides details of the structure of the data found there.

### Data description

KINECAL contains the recordings of 90 participants carrying out 11 movements, commonly used in the clinical assessment of balance impairment, frailty and falls-risk.

### Participant metadata

**register**.**csv** provides the headline information about each participant. The file contains the following columns:

**part_id:** participant id

**group:** healthy-adult (HA), non-fallers (NF), single-faller, by falls history (FHs), multiple-faller, by falls history (FHm)

**age:** chronological age at the time of recording.

**sex:** biological sex (m/f)

**height:** height in meters

**weight:** weight in kg

**BMI:** Kg/m^2^

**recorded_in_the_lab:** indicates if the recording was made in the lab

**clinically-at-Risk:** indicates if the participants were marked as clinically at risk

### Sway metrics

**sway_metric**.**csv** contains common sway metrics for each movement, per participant. This file includes the following headers:

**part_id:** participant ID

**movement:** the movement that was recorded

**group:** non-faller, single-faller, by falls history (FHs), multiple-faller by falls history (FHm)

**age:** Chronological age

**sex:** Biological sex

**recorded_in_the_lab:** indicates if the recording was made in the lab

**clinically-at-Risk:** Indicates if the participants were marked as clinically at risk

**RDIST_ML:** Value for RMS distance in ML direction

**RDIST_AP:** Value for RMS distance in AP direction

**RDIST:** Value for the overall RMS distance

**MDIST_ML**: Value for mean distance in ML direction

**MDIST_AP:** Value for man distance in AP direction

**MDIST:** Value for the overall mean distance

**TOTEX_ML:** Value for total exertion in ML direction

**TOTEX_AP:** Value for total exertion in AP direction

**TOTEX:** Value for the overall total exertion

**MVELO_ML**: Value for mean velocity in ML direction

**MVELO_AP:** Value for mean velocity in ML direction

**MVELO:** Value for the overall mean velocity

**MFREQ_ML:** Value for mean frequency in ML direction

**MFREQ_AP:**Value for mean frequency in AP direction

**MFREQ:** Value for the overall mean frequency

**AREA_CE:** The area of the 95% confidence ellipsis

### Depth data

A file has been created for each depth frame recorded by the Kinect camera (30 fps). The file stores the ushort values in binary format. The file is named DepthUshort < clock_tick > .bin. The binary file is a flattened 2D array of pixel-by-pixel distances from the camera to objects in the frame. This array can be resized back to the original 424, 512 aspect to reconstitute the original image. See the usage notes for Python code that can achieve this.

### Skeleton joint coordinates

In addition to raw depth data, the joint positions as defined by the Kinect 25 joint model has also been stored (30 fps). These are stored in plain text. The files are named < clock_tick > .txt. The file format is as follows:

**Joint_name:** The name for an individual joint, e.g. **SpineBase**

**Tracked:** This indicates if the joint was tracked or inferred. Inferred positions happen in the case of occlusion

**x-3D:** The estimated x position in 3D space

**y-3D:** The estimated x position in 3D space

**z-3D**: The estimated x position in 3D space

**x-pixel-pos**: The x-pixel potion from the depth camera

**y-pixel-pos:** The y-pixel potion from the depth camera

### Folder structure

**Fig. 6:**
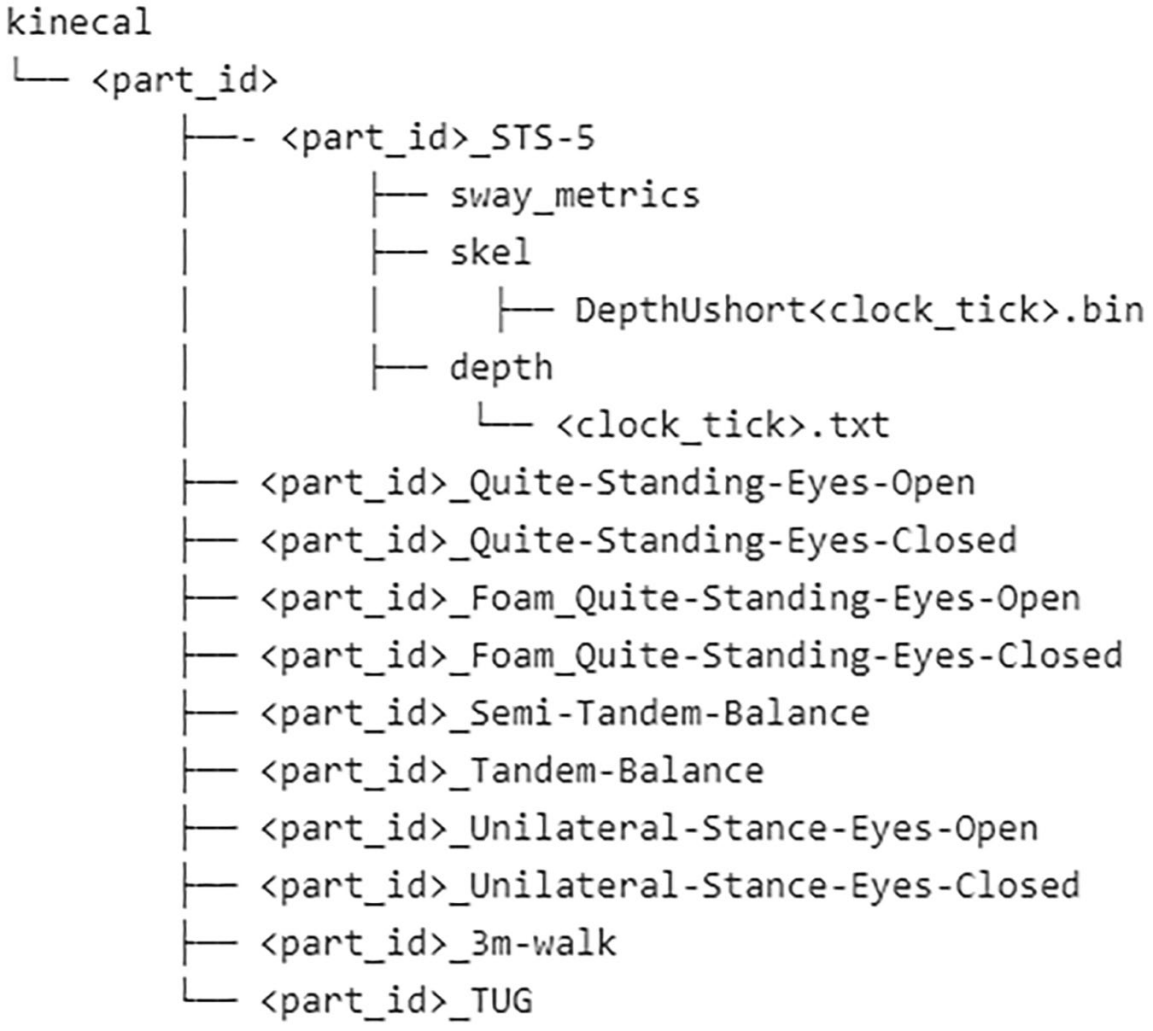
File structure for KINECAL dataset.

This section provides details of the files and folders which make up the dataset. Figure 6 shows the folder structure for the STS-5 movement. Similar folder structures exist for the other movements. They are not repeated here, for brevity’s sake.

## Technical Validation

The recordings were undertaken as laid out in the Data Collection section.

After recording, a video was created from the movements of the extracted joints, over time. Each frame represents the 3D coordinates in space for one time-step. the videos were then viewed to ensure all recordings are free of artefacts. Any videos with artefacts were excluded.

**Fig. 7:**
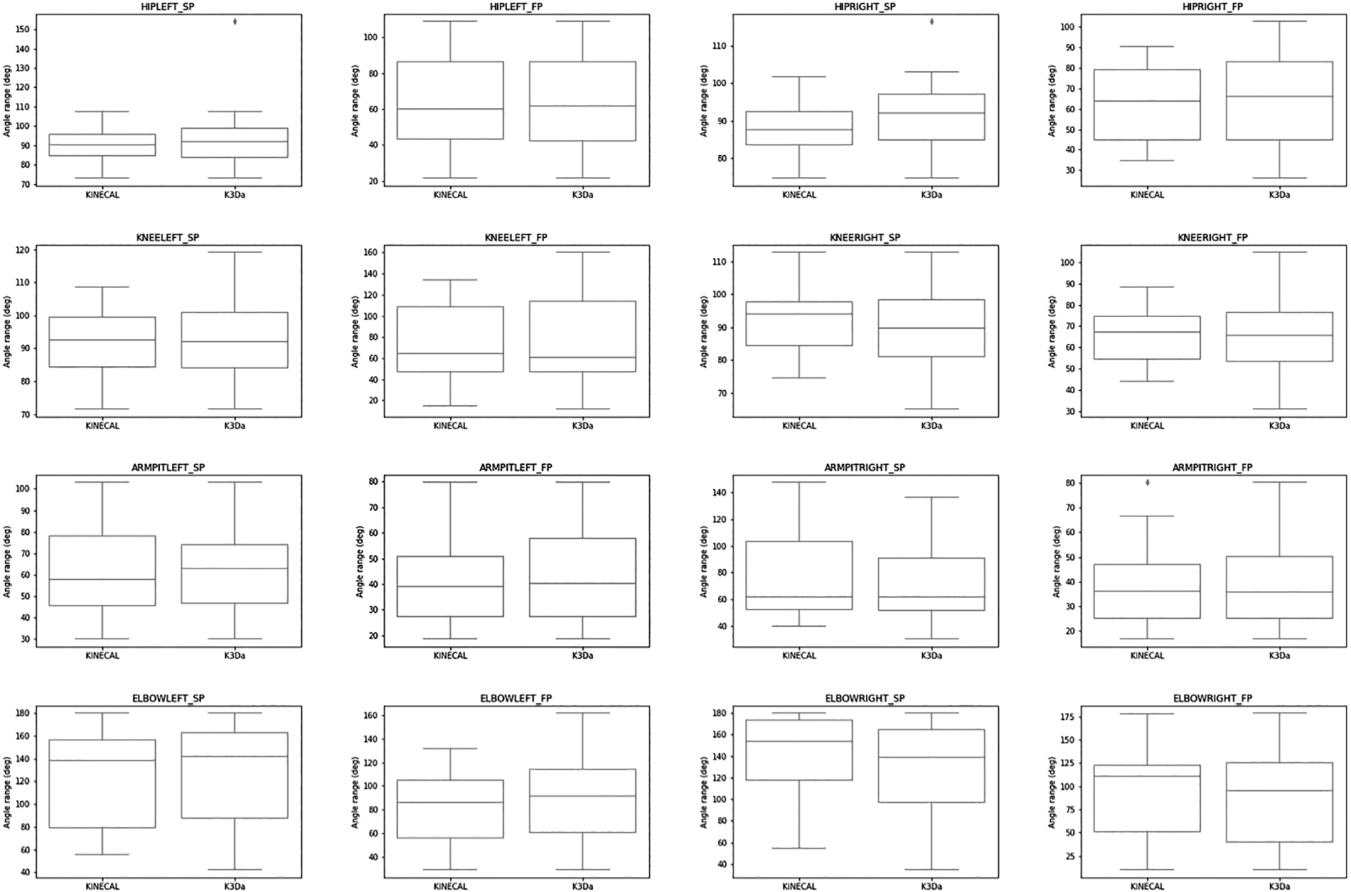
Box and whisker plots for STS-5 movement of the KINECAL and K3Da datasets: The range of movement in each joint is shown for the sagittal and frontal planes. similar ranges are seen between the two datasets. Independent t-tests were used to demonstrate no significant difference between the two datasets, shown in Table 10.

KINECAL represents a unique dataset, and as such, it is difficult to compare to other datasets. However, the previously published K3Da dataset does share similar movements. Using this dataset as ground truth, we compared the range of movement (ROM) for STS-5, in both the sagittal and frontal planes. In the comparison, we examined the healthy adult group in KINECAL and the participant’s ages < 65 years old in K3Da. The results are shown in Fig. 7 and Table 10. The KINECAL data compares very well to the K3Da data.

**Table 10:** Comparison of the STS-5 movement from KINECAL and K3Da: The table shows a comparison of KINECAL and K3Da data. the p-value shown is from an independent t-test, and in all cases indicates no significant difference between the range of movement recorded by both datasets.

Joint Angle	Range deg (Mean (SD))		p
	KINECAL	K3Da	
HIPLEFT_SP	89.74 (9.4)	92.73 (14.89)	0.46
HIPLEFT_FP	63.58 (25.9)	62.8 (24.41)	0.92
KNEELEFT_SP	91.6 (10.61)	92.38 (11.83)	0.82
KNEELEFT_FP	71.81 (36.04)	73.29 (40.37)	0.90
HIPRIGHT_SP	87.84 (6.82)	91.24 (8.83)	0.18
HIPRIGHT_FP	63.03 (19.12)	63.92 (20.68)	0.89
KNEERIGHT_SP	92.72 (10.33)	90.27 (12.04)	0.49
KNEERIGHT_FP	64.57 (11.76)	65.42 (15.24)	0.84
SPINEMID_SP	6.77 (1.34)	6.66 (1.16)	0.76
SPINEMID_FP	1.26 (0.46)	1.32 (0.51)	0.71
NECK_SP	24.09 (5.29)	24.87 (5.33)	0.63
NECK_FP	9.66 (2.81)	9.29 (2.73)	0.65
ARMPITLEFT_SP	63.61 (21.02)	63.37 (20.05)	0.97
ARMPITLEFT_FP	40.54 (16.07)	44.48 (17.1)	0.44
ARMPITRIGHT_SP	78.32 (35.24)	71.16 (29.23)	0.46
ARMPITRIGHT_FP	37.94 (16.71)	38.67 (16.12)	0.89
ELBOWLEFT_SP	122.28 (44.97)	126.9 (44.84)	0.73
ELBOWLEFT_FP	82.69 (29.22)	91.85 (32.46)	0.34
ELBOWRIGHT_SP	137.97 (39.73)	125.16 (47.39)	0.35
ELBOWRIGHT_FP	92.76 (49.69)	89.78 (52.2)	0.85

## Usage Notes

Both raw depth and joint positions are provided. These files can be processed using many commonly used data processing tools, e.g. Python and Matlab.

The data can be used to study both postural sway metrics, akin to data derived from force plates and whole body metrics, such as joint angles, akin to metrics derived from marker-based motion capture, such as VICON. Sample code is available^37^ to demonstrate how this is achieved.

## Data Availability

The code used to process the files, along with some example scripts are available from a physionet repository^37^.
